# Editorial: Insights in lipids, membranes and membranous organelles

**DOI:** 10.3389/fmolb.2023.1210167

**Published:** 2023-05-03

**Authors:** Jin Ye

**Affiliations:** Department of Molecular Genetics, University of Texas Southwestern Medical Center, Dallas, TX, United States

**Keywords:** membranes, lipids, lipid protein interaction, membrane protein, ERAD (ER associated protein degradation), protein carbonylation

The boundary of a cell and subcellular organelles within it are defined by membranes, which are composed of lipids and proteins. This Research Topic illustrates the critical importance of lipid-protein interactions in targeting and regulation of membrane proteins. Membrane proteins are categorized into peripheral-associated and intrinsic transmembrane proteins. For peripheral membrane proteins, their localization on membranes is primarily determined through their interaction with the hydrophilic head groups of phospholipids or other lipids on membrane surface. In this Research Topic, Peters et al. and Capelluto et al. depict the structural details of the interaction between these proteins and membranes containing phosphatidylserine or sulfatide, a glycosphingolipid that contains a sulfate moiety in the hydrophilic head group. In contrast, the interaction between intrinsic transmembrane proteins and hydrophobic interiors of membranous lipids is critical for insertion of these proteins into membranes. Nishikawa et al. in this Research Topic illustrate how a glycolipid in *E. Coli* facilitates this interaction to catalyze integration of these proteins into membranes.

The localization of some proteins on membranes is more important for their regulations than functions. One such example is 3-hydroxy-3-methyl-glutaryl-coenzyme A reductase (HMGCR), which catalyzes the rate limiting step in cholesterol biosynthesis pathway for production of sterols and nonsterol isoprenoids (Goldstein and Brown, 1990). HMGCR contains a cytosolic catalytic domain at the C-terminus and a membrane-anchoring domain at the N-terminus. The catalytic domain by itself is completely functional without association with membranes; However, the presence of the N-terminal domain allows the degradation of HMGCR to be regulated by lipid products of the enzyme (Gil et al., 1985). HMGCR is subject to ER-associated degradation, a two-step process consisting ubiquitination followed by extraction of the ubiquitinated proteins from ER membranes to cytosol for proteasomal degradation (Oikonomou and Hendershot, 2020). In this Research Topic, Faulkner and Jo illustrate how sterols and a nonsterol isoprenoid accelerate degradation of HMGCR at the ubiquitination and extraction step, respectively.

While the regulation of HMGCR by lipids is important for normal cell physiology, other protein-lipid interactions may have pathological consequences. One such example is protein carbonylation in diseases such as Alzheimer’s disease that is associated with oxidative stress (Manoharan et al., 2016), which leads to peroxidation of polyunsaturated fatty acids. The breakdown of lipid peroxidation products generates reactive aldehydes such as 4-hydroxy-2-nonenal (HNE), which covalently modifies proteins nonenzymatically through protein carbonylation (Fritz and Petersen, 2011). In this Research Topic, Yamashima et al. describe how HNE-modified Hsp70.1 leads to lysosomal membrane damage.

In summary, the Research Topic of articles in this Research Topic is variation of a theme on lipid-protein interactions. Publication of this Research Topic, hopefully, will stimulate further research in this interesting field.
